# Toxicity of Rocket Fuel Derivatives to the *Tenebrio molitor*–*Gregarina* spp. Host–Parasite System at Different Temperatures

**DOI:** 10.3390/biology15191700

**Published:** 2026-09-24

**Authors:** Denis Rybalka, Viktor Brygadyrenko

**Affiliations:** 1 Department of Biodiversity and Ecology, Oles Honchar Dnipro National University, 72 Nauky Ave., 49045 Dnipro, Ukraine; denisrybalka89@gmail.com; 2 Department of Parasitology and Veterinary and Sanitary Expertise, Dnipro State Agrarian and Economic University, 25 Serhiia Yefremova St., 49000 Dnipro, Ukraine

**Keywords:** hydrazine hydrate, 2,4-dinitrophenylhydrazine, 2,4-dinitroaniline, pupation, body mass, sublethal effects, soil contamination, armed conflict, terrestrial invertebrates, Coleoptera

## Abstract

Hydrazine (N_2_H_4_) is a component of rocket fuel, and its dinitroaromatic relatives are feedstocks for explosives and pesticides. Both classes contaminate soils around aerospace facilities and in areas of armed conflict. The toxicity of hydrazine hydrate, 2,4-dinitrophenylhydrazine (DNPH) and 2,4-dinitroaniline to terrestrial invertebrates remains unexplored. This study examined the effects of these substances on the mortality, body mass, pupation, and intensity of *Gregarina* spp. infestation in larvae of *Tenebrio molitor* Linnaeus, 1758 (Coleoptera, Tenebrionidae). Mortality rose with dose for all three compounds and at 90 mg/kg reached 26.7%. Pupation responded to the fact of exposure itself: none was recorded in the control (0.0%), whereas 59.6% of the exposed larvae pupated, already at the lowest concentration of 10 mg/kg, where no deaths occurred. The proportion pupating was similar across the three compounds (55.0–62.2%) but declined with increasing concentration, from 67.2% at 10 mg/kg to 52.2% at 90 mg/kg. Within the 21–28 °C range, no main effect of temperature on mortality, pupation, or body mass was found. All three compounds reduced the intensity of *Gregarina* spp. infestation with increasing concentration. Under acute 5-day exposure, therefore, the studied compounds cause little larval mortality but alter pupation, body mass and the intensity of parasitic infestation.

## Simple Summary

Hydrazine and its derivatives are rocket fuel-related pollutants that can contaminate soils near aerospace facilities and military sites. This study investigated how hydrazine hydrate, 2,4-dinitrophenylhydrazine (DNPH), and 2,4-dinitroaniline affect mealworm larvae (*Tenebrio molitor*) and their intestinal parasites (*Gregarina* spp.) in two temperature ranges. Exposure to these compounds caused only low larval mortality, even at the highest tested concentration. However, the pollutants strongly affected physiological traits, inducing early pupation and reducing body mass at concentrations that caused no deaths. The number of *Gregarina* parasites decreased as pollutant concentrations increased, suggesting that these compounds influence host–parasite interactions. Temperatures between 21 and 28 °C had little effect on mortality, pupation, or body mass, although higher temperatures reduced parasite abundance. The results indicate that the *T. molitor–Gregarina* system may serve as a sensitive bioindicator for the early detection of hydrazine contamination in soils.

## 1. Introduction

Hydrazine (N_2_H_4_) and its derivatives, particularly unsymmetrical dimethylhydrazine (UDMH), are high-energy liquid fuels widely used in the global aerospace industry, and their transformation products in soil include dimethylamine, N,N-dimethylformamide and other harmful compounds [1]. This fuel is used in both military and civilian systems [2]. Launch vehicle accidents cause local soil contamination with hydrazine compounds, which decompose within a few weeks depending on the soil type [1]. Toxicity has been recorded across distant taxa, in each case with its own exposure route and endpoint. In aquatic biocenoses, hydrazine is toxic to invertebrates and fish: the 48 h EC_50_ in *Daphnia pulex* Leydig, 1860 is 0.16–0.19 mg/L, and the 96 h LC_50_ in *Pimephales promelas* Rafinesque, 1820 is 5.98 mg/L [3]. In soil nematodes, a 1% solution of hydrazine hydrate killed more than 80% of larvae after 24 h of exposure, and 2,4-dinitrophenylhydrazine also showed a nematocidal effect [4]. According to Rybalka and Brygadyrenko [5], contact toxicity for terrestrial beetles is LC_50_ = 0.015–0.035 g/m^2^ in *T. molitor* and 0.085 g/m^2^ in *Opatrum sabulosum* (Linnaeus, 1761).

Military activity and conflicts contaminate soils with munition and explosive residues, which accumulate in the environment and resist natural degradation [6]. Concentrations of energetic compounds measured in soils at military ranges span from trace levels to tens of grams per kilogram: near a bomb crater, trinitrotoluene (TNT) ranged from 0.12 to 778 mg/kg, with a mean of 31.8 mg/kg; at ammunition burn pads, 2,4-dinitrotoluene reached 491 mg/kg; 2 m behind a firing line, nitroglycerin reached 6560 mg/kg; and in soil inside a crater from a 500 lb bomb, TNT was 42,200 mg/kg [7]. The distribution and persistence of explosives and propellants in soils are determined by the physicochemical properties of the compounds and the characteristics of the soil environment [7]. Organic pollutants of military origin affect soil microbial communities, nutrient cycling and plant physiology through oxidative stress, bioaccumulation and growth inhibition [8]. Explosions of strike unmanned aerial vehicles also alter soil acidity: at explosion sites, active acidity was 6.62–7.41 with a mean of 7.14, which the authors attribute to carbonate parent rock released from the blast crater [9]. Accidents involving Proton-M launch vehicles in Kazakhstan between 1999 and 2018 caused soil contamination with UDMH at concentrations exceeding regulatory limits [10]. At rocket-stage fall sites, the UDMH content of soil reaches 240 mg/kg near the center of the crater, against a maximum permissible concentration of 0.1 mg/kg, and soil respiration is fully suppressed above 200 mg/kg [11]. The armed conflict in Ukraine (since 2014) is creating a new scale of soil contamination with rocket fuel components due to the intensive use of missile systems [12,13,14].

Hydrazine toxicity arises from oxidative stress with the production of reactive oxygen species, formation of DNA adducts (N^7^-methylguanine and O^6^-methylguanine), and depletion of pyridoxine (vitamin B_6_), which inhibits the synthesis of gamma-aminobutyric acid (GABA) and causes neurotoxic effects [15,16]. Hydrazine causes mitochondrial dysfunction and oxidative damage that leads to hepatotoxicity [17]. This compound is a carcinogen in mammals [18] and is classified by the International Agency for Research on Cancer as probably carcinogenic to humans, Group 2A [16].

The compounds under investigation—hydrazine hydrate (N_2_H_4_·H_2_O), 2,4-dinitrophenylhydrazine (DNPH), and 2,4-dinitroaniline—form a structural series (Figure 1) ranging from the parent hydrazine to its aromatic derivative to a related aromatic amine. Hydrazine hydrate is used as rocket fuel. DNPH combines a hydrazine functional group with a dinitrophenyl moiety and serves as a raw material in the industrial production of explosives [19]. 2,4-Dinitroaniline is a component of dinitroaniline herbicides that are toxic to soil and aquatic invertebrates [20]. The contact toxicity of these compounds for *T. molitor* increases in the same order [5].

Temperature influences the toxicity of pollutants by accelerating metabolic processes through increased rates of enzymatic reactions, enhancing the permeability of cell membranes, and intensifying oxidative stress [22]. In invertebrates, as poikilothermic organisms, temperature changes determine the dynamics of metabolism and detoxification. The interpretation of toxicity under different temperature regimes is therefore important for predicting ecological risks. The temperature dependence of the toxicity of hydrazine compounds requires experimental verification, as the available data are limited to aquatic organisms and mammals.

*Tenebrio molitor*, the mealworm beetle (Coleoptera, Tenebrionidae), is used in toxicological studies as a simplified model animal organism, in accordance with the 3R principles (Replacement, Reduction, Refinement) of the ethical use of animals, which ensures cost-effectiveness and rapid testing [23]. It is the most common insect species for rearing in Europe, and the number of scientific publications on it has grown intensively following the adoption of EU Regulations 2283/2015 and 2017/893 [24]. Previous studies have confirmed the sensitivity of *T. molitor* to organic xenobiotics of various classes [25,26]; however, the effect of hydrazine derivatives on this model organism remains unexplored.

The intensity of parasitic infestation is a sensitive indicator of ecosystem status. Gregarines are the most common parasites of *T. molitor*, and the prevalence of parasites in cultivated edible insects gives the study of this system applied significance for food safety [27]. The literature describes *T. molitor* larvae as hosts to three species of gregarines: *Gregarina cuneata* Stein, 1848, *G. polymorpha* (Hammerschmidt, 1838), and *G. steini* Berndt, 1902. A molecular check of this list recovered mainly *G. polymorpha*, and no trophozoites with the features of *G. steini* were found [28]. The intensity of gregarine infestation is related to the size and growth rate of the host larvae: smaller larvae carried fewer gregarines, and a positive relationship was found between mass gain and infestation intensity [25]. Chemical stress affects host–parasite relationships: sublethal concentrations of insecticides increase the abundance of gregarines by suppressing the host’s defensive responses [29]. The physiological state of the host also influences gregarine infestation, particularly under starvation [30].

The available data on the toxicity of hydrazine compounds concern mainly aquatic organisms and mammals, and for terrestrial invertebrates, they are limited to contact exposure [5]. Substrate exposure, in which compounds enter an organism with food, and the temperature dependence of this toxicity in a host–parasite context remain unexplored. Given the scale of soil contamination around aerospace facilities and military conflict zones [10,13], this problem requires more detailed analysis.

This study aims to determine the effect of three compounds (hydrazine hydrate, DNPH, 2,4-dinitroaniline) on the mortality, body mass, pupation, and intensity of *Gregarina* spp. infestation in *T. molitor* larvae at two temperatures (21–23 and 26–28 °C). The hypotheses were that (1) a temperature of 26–28 °C, compared with 21–23 °C, would enhance the toxic effects of the studied compounds through accelerated metabolic processes; (2) sublethal endpoints—pupation, body mass, and intensity of parasitic infestation—would respond at concentrations causing no mortality; and (3) toxicity would increase according to the position of the compound in the structural series (hydrazine hydrate < DNPH < 2,4-dinitroaniline), as established for contact toxicity.

## 2. Materials and Methods

### 2.1. Animals and Housing

*Tenebrio molitor* larvae were kept in transparent plastic containers (40 × 30 × 20 cm) at a temperature of 21–23 or 26–28 °C (depending on the experimental temperature regime), a relative humidity of 40–50%, and indirect sunlight. The feeding substrate consisted of organically produced pressed oat grains (2025 harvest). The water source for *T. molitor* was cabbage leaves, which were placed on the surface of the feeding substrate after being cut into 1 × 1 cm pieces. They were supplied identically to every group, including the controls, as in the routine rearing of this species. Each larva in the container was provided with 0.30 ± 0.03 g of cabbage leaves. The leaves were replaced every 2 days to prevent mold growth. Humidity and lighting were standardized for all groups within each temperature regime and between the regimes. All larvae had equal access to food and the same likelihood of parasitic infestation.

For the experiment, 600 *T. molitor* larvae 18–22 mm in length, without sex determination, were selected from a laboratory colony of more than 4000 larvae, which made it possible to choose individuals within a narrow length window and so standardize body size across groups. Larvae of this species usually reach 20–35 mm in length [31], so the individuals selected here fell in the lower part of that range and were in the last larval instar. Inclusion criteria were defined a priori in accordance with the Animal Research: Reporting of In Vivo Experiments (ARRIVE) 2.0 guidelines [32]: larvae 18–22 mm in length with no visible signs of disease or damage. Selection was based on body length alone, so the instar number, the age of the larvae, and the time since the last molt remained undetermined. The mean initial body mass was 144.4 ± 22.5 mg/individual.

### 2.2. Experimental Design and Treatment Groups

Three hundred larvae were randomly assigned to each of the two temperature regimes (21–23 and 26–28 °C) to assess their effect on toxicity. At each temperature, the larvae were divided into ten treatment groups: a control group (without the addition of compounds) and nine groups exposed to three concentrations (10, 30, 90 mg/kg substrate) of three compounds (hydrazine hydrate, DNPH, 2,4-dinitroaniline). Each treatment group comprised 30 larvae at each temperature regime, held as three containers of 10 larvae rather than as one group of 30. The containers were transparent plastic, 500 mL in volume, and each held 50 ± 1 g of pressed oat grains. Across the ten treatment groups and the two temperature regimes, this gave 60 containers and 600 larvae in total. They were arranged in a randomized complete block design with three blocks, each holding one container of every treatment group. Positions within a block were determined using a random number generator in accordance with the ARRIVE 2.0 guidelines [32]. Because the compounds were added to the substrate of the container rather than to individual larvae, the container served as the experimental unit.

### 2.3. Chemicals and Exposure

All chemicals were purchased from Sigma-Aldrich Chemie GmbH (Taufkirchen, Germany) and prepared as standard stock solutions. The compounds were dissolved in dimethyl sulfoxide (DMSO) to ensure a homogeneous distribution in the substrate. All concentrations reported are nominal: substrate concentrations were not verified analytically at the start or the end of the exposure. The DMSO concentration was selected on the basis of preliminary testing in which the LC_50_ of the solvent was 0.964 ± 0.196 g/m^2^ [5], so as to avoid background effects on the larvae. Control groups received an equivalent amount of DMSO without the tested compounds. The solutions were evenly applied to the feed substrate using a pipette to a depth of 20 mm from the surface. The substrate was thoroughly mixed, after which the larvae were placed in it. Each larva was marked with an individual number for tracking.

Concentrations (10, 30, 90 mg/kg substrate) and sample size were determined based on a pilot experiment, statistical power analysis, our earlier determination of the contact LC_50_ of these three compounds for this species [5], and reported soil concentrations at aerospace and military sites [10]. A preliminary study on the acute contact toxicity of these compounds to *T. molitor* larvae [5] confirmed the species’ sensitivity to hydrazine hydrate, DNPH, and 2,4-dinitroaniline (LC_50_: 0.035 ± 0.006, 0.026 ± 0.004, and 0.015 ± 0.001 g/m^2^, respectively). A pilot experiment with n = 50 larvae (5 per compound–concentration combination, 5 in the control, temperature 21–23 °C) showed body mass loss under substrate exposure in every treatment group, whereas control larvae gained mass (Table S1, Supplementary Materials; the dataset is also in the repository cited in the Data Availability Statement). Effect sizes (Cohen’s d) for body mass changes ranged from 2.07 to 3.32 depending on the compound and concentration, with a median value of d = 2.78, which corresponds to a large effect by Cohen’s criteria [33]. At that effect size, n = 4 larvae per group provides a statistical power of 0.80 at *p* = 0.050. The sample size was increased to n = 30 larvae per treatment group to account for mortality and the exclusion of individuals that pupated from the analyses of body mass and intensity of parasitic infestation. The concentrations were chosen to span the range from no mortality to a measurable lethal effect, so that sublethal responses could be separated from lethal ones.

The experiment lasted 5 days, during which the larvae were exposed to the chemicals through both contact with and ingestion of the treated substrate. Food consumption was not measured, so the relative contribution of the two routes was not determined. Control groups were kept under the same conditions but without the addition of toxicants. A cotton disc was placed at the bottom of each plastic container to absorb excess moisture. After the 5-day exposure, mortality was recorded, and each *T. molitor* larva was weighed individually to assess changes in body mass. Exclusion criteria were defined a priori. Dead larvae were left out of the analysis of body mass and intensity of parasitic infestation. Individuals that pupated were also excluded from the dose–response analyses of these two parameters, as metamorphosis causes mass loss independent of toxic effects and distorts the assessment of the dose-dependent response [32]. Their measurements were retained and used in a separate comparison of body mass changes between individuals that pupated and those that remained as larvae, which addresses a different question. Individuals that pupated were not dissected, so no gregarine counts exist for them, and the parasite results therefore describe surviving larvae that did not pupate. Metamorphosis also ends the larval infection. The three *Gregarina* species examined here parasitize the larval gut and do not establish in adult beetles, whose guts are reorganized during holometabolous development [34].

### 2.4. Parasitological Examination

Immediately after the end of the 5-day exposure, on the same day as weighing, the surviving *T. molitor* larvae were dissected for parasitological examination. The midgut (from the stomach to the Malpighian tubules) was placed on a microscope slide in a drop of physiological saline (0.9% NaCl) to prevent drying. The intestine was opened with a scalpel, making 12 transverse incisions with particular attention to the posterior part of the midgut, where *Gregarina* spp. trophozoites are localized [35]. Immature gregarines still attached to the intestinal wall by an epimerite were not counted; such stages are embedded in the epithelium, and their epimerites are ruptured during squash preparation [36]. Only trophozoites released from the midgut during standardized compression with a cover slip were counted. Parasite counts were performed using a blind method: samples were coded with random numbers before dissection, which, together with the microscopic counting, was carried out by two trained researchers who had no access to the group membership of the sample, and the code was broken only after all the work was completed [32]. *Gregarina cuneata*, *G. polymorpha*, and *G. steini* were identified from morphological characteristics following Valigurová [36], Nocciolini et al. [28], and Desportes and Schrével [37].

### 2.5. Statistical Analysis

Each endpoint was analyzed with a model suited to its distribution: a linear mixed model for body mass, binomial mixed models for mortality and pupation, and a zero-inflated negative binomial mixed model for parasite counts. Every model included container as a random effect.

Body mass changes were analyzed using a linear mixed model (LMM) with temperature, treatment group, and their interaction as fixed effects, initial body mass as a covariate, and container as a random effect, with degrees of freedom estimated by the Satterthwaite method. Differences between each treatment group and the control were estimated as Dunnett contrasts with 95% confidence intervals and standardized as Cohen’s d relative to the residual standard deviation. The letters above the boxplots were obtained from pairwise contrasts of the same model with Tukey adjustment [38].

For the analysis of binary outcomes (mortality, pupation), binomial generalized linear mixed models (GLMMs) with a logit link function and container as a random effect were used. The statistical significance of the main effects was determined using likelihood ratio tests comparing the full model with the model lacking the corresponding predictor. The threshold nature of the pupation response was examined separately: the concentration effect among the exposed larvae was assessed with a mixed model, and the difference between the control and treatment groups was assessed with Fisher’s exact test.

The total number of parasites (the sum of *G. cuneata*, *G. polymorpha*, and *G. steini*) was analyzed using a zero-inflated negative binomial mixed model (ZINB) with container as a random effect. Each species was then modeled separately with the same structure to establish which of them carried the effect observed in the total. For *G. cuneata*, the rarest of the three, the zero-inflated model did not converge, and this species was fitted without zero inflation. Four types of distributions were compared: Poisson, negative binomial (NB), zero-inflated Poisson (ZIP), and ZINB. The optimal specification was chosen by the Akaike Information Criterion (AIC), where lower values indicate a better fit to the data. The ZINB model showed the lowest AIC values for all three compounds, so one specification served the whole analysis, consistent with typical characteristics of parasitological data: overdispersion and structural zeros.

Statistical analysis was performed in R version 4.5.1 [39] using the following packages (versions in parentheses): dplyr (1.1.4) for data processing, ggplot2 (4.0.1) and patchwork (1.3.2) for visualization, tidyr (1.3.1) for data reshaping, lme4 (1.1.37) and lmerTest (3.2.1) for linear and generalized mixed models, glmmTMB (1.1.14) for zero-inflated models, emmeans (1.11.2.8) and multcomp (1.4-28) for contrasts and compact letter display, MASS (7.3-65) and pscl (1.5.9), described by Zeileis et al. [40], for distribution comparison, car (3.1-3) for Levene’s test, and pwr (1.3-0) for power analysis.

## 3. Results

### 3.1. Mortality of Tenebtio molitor Larvae

Larval mortality increased with increasing concentrations of the tested compounds (Figure 2). No mortality was recorded in the control groups (0 mg/kg substrate) at either temperature (21–23 and 26–28 °C). At the maximum concentration of 90 mg/kg, mortality was 13.3% and 26.7% for DNPH at 21–23 and 26–28 °C, respectively, and 10.0% for 2,4-dinitroaniline and 6.7% for hydrazine hydrate at both temperatures. In the treatment groups, mortality was 7.4% at 26–28 °C and 4.4% at 21–23 °C (n = 270 per temperature group, excluding controls).

A binomial GLMM revealed an effect of concentration and compound on mortality (χ^2^ = 28.29, df = 3, *p* = 3.15 × 10^−6^), whereas the effect of temperature was not significant (χ^2^ = 2.31, df = 1, *p* = 0.129). The odds of death increased with concentration for all three compounds: each additional 10 mg/kg substrate multiplied them by 1.19 to 1.41 depending on the compound. Estimates with 95% confidence intervals are given in Table 1. Variability among containers was virtually zero, indicating an absence of clustering of mortality within them.

### 3.2. Pupation of Tenebrio molitor Larvae

Exposure to the studied compounds induced pupation of *T. molitor* larvae (Figure 3). No pupation was recorded in the control groups, whereas 59.6% of the exposed larvae pupated. Fisher’s exact test confirmed the statistical significance of pupation induction compared with the control (*p* = 1.53 × 10^−24^). Proportions for the individual compounds are given in Table 2.

Among the exposed larvae, the proportion that pupated declined with increasing concentration (67.2% at 10 mg/kg, 59.4% at 30 mg/kg, and 52.2% at 90 mg/kg; n = 180 per concentration). A binomial GLMM confirmed this concentration effect (χ^2^ = 6.62, df = 1, *p* = 0.010). The pupation frequency was 57.8% at 21–23 °C and 61.5% at 26–28 °C (n = 270 per temperature group). No effect of temperature was detected (χ^2^ = 0.71, df = 1, *p* = 0.398).

Initial body mass and its daily changes did not differ between the larvae that pupated and those that remained as larvae (mass: 147.8 ± 20.7 vs. 147.3 ± 21.5 mg/larva, t = 0.295, df = 373.5, *p* = 0.768; change: −3.15 ± 1.82 vs. −3.10 ± 1.75 mg/larva per day, t = −0.326, df = 400, *p* = 0.745).

### 3.3. Body Mass Change in Tenebrio molitor

The linear mixed model (larvae that did not pupate, n = 246) revealed an effect of treatment group on daily body mass changes (F = 40.57, df = 9, 40.02, *p* < 2.2 × 10^−16^). No main effect of temperature was recorded (F = 0.13, df = 1, 42.52, *p* = 0.716), but its interaction with treatment group was statistically significant (F = 2.32, df = 9, 37.52, *p* = 0.034). Initial body mass contributed to the model (F = 10.73, df = 1, 224.22, *p* = 0.001): lighter larvae gained mass more rapidly over the exposure period. The standard deviation of the container random effect was 0.562 against a residual of 1.431 mg/larva per day.

Compared with the control, all nine treatment groups reduced daily body mass changes (all *p* < 0.001). The reduction ranged from −3.86 to −7.53 mg/larva per day and corresponded to large effect sizes (d from −2.69 to −5.26). The extremes of that range belong to the groups at 90 mg/kg: −7.53 mg/larva per day for 2,4-dinitroaniline and −3.86 mg/larva per day for DNPH. Full estimates with 95% confidence intervals are given in Table 3.

Pairwise comparisons within the same model (with Tukey adjustment) separated the groups similarly for most compounds (Figure 4, Figure 5 and Figure 6). For hydrazine hydrate and 2,4-dinitroaniline at both temperatures, the control separated from all three concentrations, which did not differ from one another. For DNPH at 21–23 °C, the pattern was the same, whereas at 26–28 °C, the maximum concentration (90 mg/kg) additionally separated from the lower ones. In this group, no mass loss was recorded: the mean was +0.48 mg/larva per day, which produced the statistically significant interaction between concentration and temperature.

### 3.4. Intensity of Gregarina spp. Infestation

The zero-inflated negative binomial mixed model revealed a decrease in the total number of parasites (the sum of *G. cuneata*, *G. polymorpha*, and *G. steini*) with increasing concentration for all three compounds, as shown in Figure 7: hydrazine hydrate (z = −3.17, *p* = 0.002), DNPH (z = −2.34, *p* = 0.019), and 2,4-dinitroaniline (z = −2.26, *p* = 0.024). The concentration slopes did not differ between compounds (χ^2^ = 0.10, df = 2, *p* = 0.949). The mean number of parasites decreased from 21.2 in the control to 16.7, 13.5, and 11.7 specimens/larva at concentrations of 10, 30, and 90 mg/kg, respectively. Temperature also affected the intensity of parasitic infestation (z = −2.25, *p* = 0.024): at 26–28 °C, the mean number of parasites was 13.0 versus 18.0 specimens/larva at 21–23 °C.

Modeled separately, the three *Gregarina* species behaved differently (Figure 8, Figure 9 and Figure 10). The concentration effect on the total burden was carried by *G. polymorpha*, the most abundant of the three (z = −3.16, *p* = 0.002). Neither *G. cuneata* (z = −1.32, *p* = 0.187) nor *G. steini* (z = −0.78, *p* = 0.433) showed a concentration effect of its own. Mean counts of all three species were nevertheless lower at the highest concentration than in the control.

## 4. Discussion

### 4.1. Toxicity to Tenebrio molitor Larvae

The studied compounds killed few *T. molitor* larvae during the 5-day exposure. Three-quarters of the larvae survived even the worst combination of compound, dose and temperature, and at 10 mg/kg, none died at all. Lethality is thus not the endpoint on which these compounds act over five days, which is why the sublethal responses recorded at concentrations causing no deaths carry the weight of this study. The odds of death rose with concentration for each compound, with overlapping confidence intervals, so the order of contact toxicity reported for this species was not reproduced under substrate exposure.

The toxicity of hydrazine compounds to soil invertebrates remains poorly studied, and the available toxicological data concern mainly aquatic organisms: the 96 h LC_50_ of hydrazine hydrate for *P. promelas* is 5.98 mg/L, and the 48 h EC_50_ for *D. pulex* is 0.16–0.19 mg/L [3]. QSAR modeling indicates high acute toxicity of certain UDMH transformation products [1]. This study provides the first data on the substrate toxicity of hydrazine hydrate, DNPH, and 2,4-dinitroaniline to *T. molitor* larvae, demonstrating that changes in physiological parameters (pupation, body mass, and intensity of parasitic infestation) appear without larval death as low as 10 mg/kg substrate.

### 4.2. Exposure Effects on Pupation

Exposure to the studied compounds induced pupation in nearly 60% of *T. molitor* larvae. Pupation was induced by the very fact of exposure: it was absent in the control groups and common in every exposed group. However, among the exposed larvae, the pupation frequency declined with increasing concentration, indicating suppression of metamorphosis at higher doses. Pupation ceases feeding, so the reduced contact with the toxic substrate is a mechanical consequence of the transition itself rather than evidence of an adaptive function. Within the 5-day observation period, pupal survival could not be assessed independently of the shorter time that the pupae spent on the substrate, and the pupae were not tracked to adult emergence. An alternative explanation cannot be excluded: the larvae that pupated under stress may have been primarily those already closest to metamorphosis, meaning this reflects selection rather than induction.

Accelerated metamorphosis under chemical stress has been described in other Coleoptera species. Li et al. [41] suggested that oxidative stress in *Tribolium castaneum* (Herbst, 1797) may accelerate metamorphosis through an increase in the level of 20-hydroxyecdysone. Kodrík et al. [42] showed that adipokinetic hormone (AKH) is the principal insect stress response hormone, whose level rises under oxidative stress. The concept of “adaptive bailout” [43] posits that larvae that have reached a critical mass initiate premature metamorphosis under adverse conditions as a survival strategy. In this study, neither initial body mass nor daily mass change differed between individuals that pupated and those that remained as larvae, which does not support the existence of a size threshold for initiating metamorphosis under the “adaptive bailout” concept.

### 4.3. Exposure Effects on Body Mass

Control larvae gained mass, whereas all treatment groups lost mass when averaged over the two temperature regimes. The single exception was the 2,4-dinitrophenylhydrazine group at 90 mg/kg and 26–28 °C, for which no mass loss was recorded. The loss was already large at the lowest concentration and changed little with further increases: at both temperatures, the control separated from all exposed groups, which, for the most part, did not differ from one another. Mass reduction under the influence of toxic compounds is a typical response of invertebrates [23]. Hydrazine compounds may cause avoidance of the contaminated substrate through stimulation of deterrent receptors [44,45]. However, we did not measure food consumption, so we cannot separate reduced feeding from a direct toxic effect on metabolism. The mass loss is equally consistent with both mechanisms, as well as with their combination.

### 4.4. Exposure Effects on Gregarina spp. Infestation

The intensity of parasitic infestation fell as concentration rose for all three studied compounds alike, and the concentration slopes did not differ between them. This aligns the studied compounds with ammonia, which also caused a dose-dependent reduction in the intensity of parasitic infestation [46]. Lazurska and Brygadyrenko [25] showed that only four of the 24 organic xenobiotics they studied reduced the abundance of *G. polymorpha* in *T. molitor*, indicating the dependence of the parasitic response on the chemical structure of the compound. The ability to reduce gregarine infestation is therefore shared by the studied compounds and ammonia.

The mechanism underlying the reduction in the intensity of parasitic infestation may be associated with the direct toxicity of the studied compounds to *Gregarina* trophozoites in the host’s intestine or with indirect changes in the intestinal environment under exposure to the toxicant. Because the effect was similar for volatile (hydrazine hydrate) and non-volatile (DNPH, 2,4-dinitroaniline) derivatives, volatility is unlikely to be the determining factor. Temperature also affected the intensity of parasitic infestation, which was lower at the higher regime. A moderate increase in temperature probably accelerates the metabolism of the parasites and shortens their residence time in the intestine.

### 4.5. Effect of Temperature on Toxicity

No main effect of temperature on mortality or pupation was found. For body mass change, no main effect was recorded either, but temperature interacted with treatment group: it did not shift mass loss uniformly; it changed the shape of the dose-dependent response. The interaction was driven mainly by DNPH, for which mass loss vanished at the highest concentration and the higher temperature, whereas for hydrazine hydrate and 2,4-dinitroaniline, the group pattern was the same at both temperatures. Such temperature modulation may be associated with the acceleration of metabolic processes at elevated temperatures [22].

The two regimes sit differently relative to the optimum of *T. molitor*: according to Podlesnik [47], a temperature of 25–30 °C is considered optimal for development, so 26–28 °C lies within this range, while 21–23 °C lies below it. In heat stress experiments on this species, 28 °C is used as the non-stress control, and stress is induced at 38 °C [48], so the upper regime of this study corresponds to the control temperature of such work. The absence of a main temperature effect on mortality, body mass, and pupation is consistent with neither regime imposing thermal stress and with the data for ammonia, where no temperature modulation of toxicity was recorded in this range either [46]. The reduction in the intensity of parasitic infestation at the higher temperature probably reflects the accelerated metabolism of the parasites themselves rather than heat stress in the host, since both regimes lie within limits that this species tolerates without signs of stress.

### 4.6. Implications for Biomonitoring and Insect Rearing

Pupation responded to exposure to the studied compounds as low as 10 mg/kg, where no mortality was recorded. *Tenebrio molitor* is mass-reared as a sustainable source of protein for feed and human nutrition and is also used as a model organism in toxicological studies in accordance with the 3R principles [23]. The species is approved in the EU as a novel food [24]. The induction of premature pupation could serve as an indicator of substrate contamination during production, although this hypothesis requires validation. At the same time, these considerations apply only to acute 5-day exposure. We did not measure reproduction, developmental duration, adult emergence, or other chronic parameters, so the implications for industrial rearing require verification in experiments covering the full life cycle.

The study’s findings are also relevant in the context of soil contamination with hydrazine compounds resulting from military operations in Ukraine [13,49,50] and the activities of aerospace facilities, where UDMH concentrations in soils exceed regulatory limits [10]. *Tenebrio molitor* larvae could be used as bioindicators of substrate contamination with hydrazine compounds, where pupation would signal exposure and changes in body mass and intensity of parasitic infestation would quantify the degree of impact. Such use remains a hypothesis: it requires calibration against analytically measured concentrations, validation in real soils, and evidence that the response distinguishes contamination by hydrazine compounds from the action of other stressors. None of these conditions was tested in this study.

### 4.7. Limitations of the Study

This study has several limitations. The 5-day exposure reflects only acute toxicity and does not allow the assessment of chronic effects of longer exposure. Neither temperature regime (21–23 and 26–28 °C) imposes thermal stress on *T. molitor*, which limits the extrapolation of the results to extreme temperatures. A single substrate type (pressed oat grain) was used, although the bioavailability of the compounds may differ in other substrates or soils. The larvae were selected based on a body length of 18–22 mm, so a laboratory experiment of this design does not cover all larval stages and does not distinguish individual instars. Individual compounds were studied, whereas in field conditions, organisms are exposed to mixtures of UDMH transformation products [1]. Excluding larvae that pupated from the analysis of body mass and intensity of parasitic infestation reduced the sample size. Only *Gregarina* trophozoites were counted, and other stages of parasite development (cysts and gamonts) were not assessed. Dead larvae were not dissected, so at the highest concentrations, where mortality was greatest, the sample for parasitological analysis may have been biased toward the individuals that survived. We did not measure food consumption, oxidative stress, detoxification enzyme activity, or immune markers, so the proposed mechanisms remain hypotheses that require separate experimental verification. All exposure concentrations are nominal, since substrate concentrations were not verified analytically, and loss of hydrazine hydrate over the 5-day exposure cannot be excluded; comparisons between compounds therefore rest on nominal rather than measured exposure. Substrate moisture, equilibration time after application, and any solvent evaporation were likewise not recorded. Because food consumption was not measured, the compounds reached the larvae through both contact and ingestion in proportions that remain undetermined.

## 5. Conclusions

The study revealed low acute toxicity of the studied compounds (hydrazine hydrate, DNPH, 2,4-dinitroaniline) to *T. molitor* larvae under substrate exposure, and the predicted increase in toxicity according to the position of the compound in the structural series was not observed. The predicted enhancement in toxicity at the higher temperature was likewise not observed within the 21–28 °C range. Sublethal endpoints, by contrast, responded at concentrations causing no mortality: pupation, loss of body mass, and a reduction in the intensity of *Gregarina* spp. infestation. Accelerated metamorphosis may be a stress-associated response that reduces contact with the contaminated substrate, although the 5-day observation period does not allow this to be separated from the shorter time that the pupae spent on the substrate. The results are consistent with the suitability of *T. molitor* and its associated *Gregarina* spp. as a model system for assessing the toxicity of the studied compounds under acute exposure. Further studies should include chronic exposures, an expanded temperature range, and the analysis of mixtures of UDMH transformation products.

## Figures and Tables

**Figure 1:**
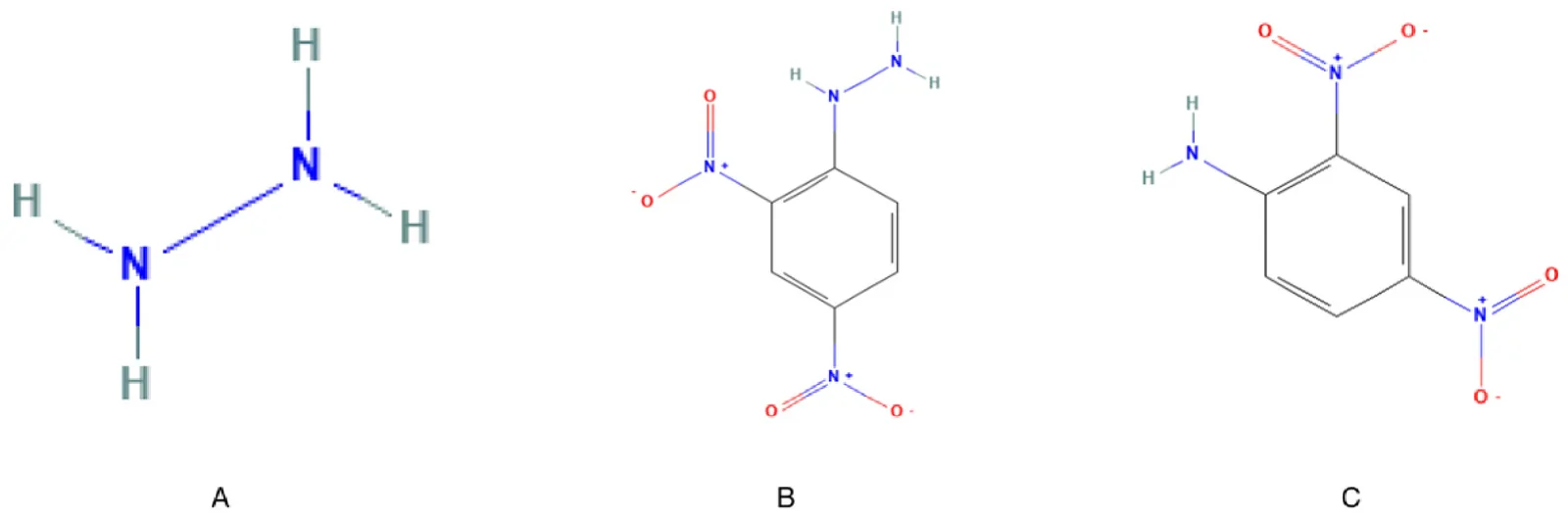
Structural formulae of the three compounds studied here, which form a structural series: (**A**)—hydrazine (N_2_H_4_), the parent compound of the series, applied in the experiment as hydrazine hydrate (N_2_H_4_·H_2_O); (**B**)—2,4-dinitrophenylhydrazine (DNPH, C_6_H_6_N_4_O_4_), an aromatic derivative of hydrazine in which the hydrazine group is joined to a dinitrophenyl moiety; (**C**)—2,4-dinitroaniline (C_6_H_5_N_3_O_4_), a related aromatic amine in which the hydrazine group is absent; atom colours follow the PubChem convention: nitrogen blue, oxygen red, carbon skeleton black; two-dimensional depictions were retrieved from the PubChem database [21].

**Figure 2:**
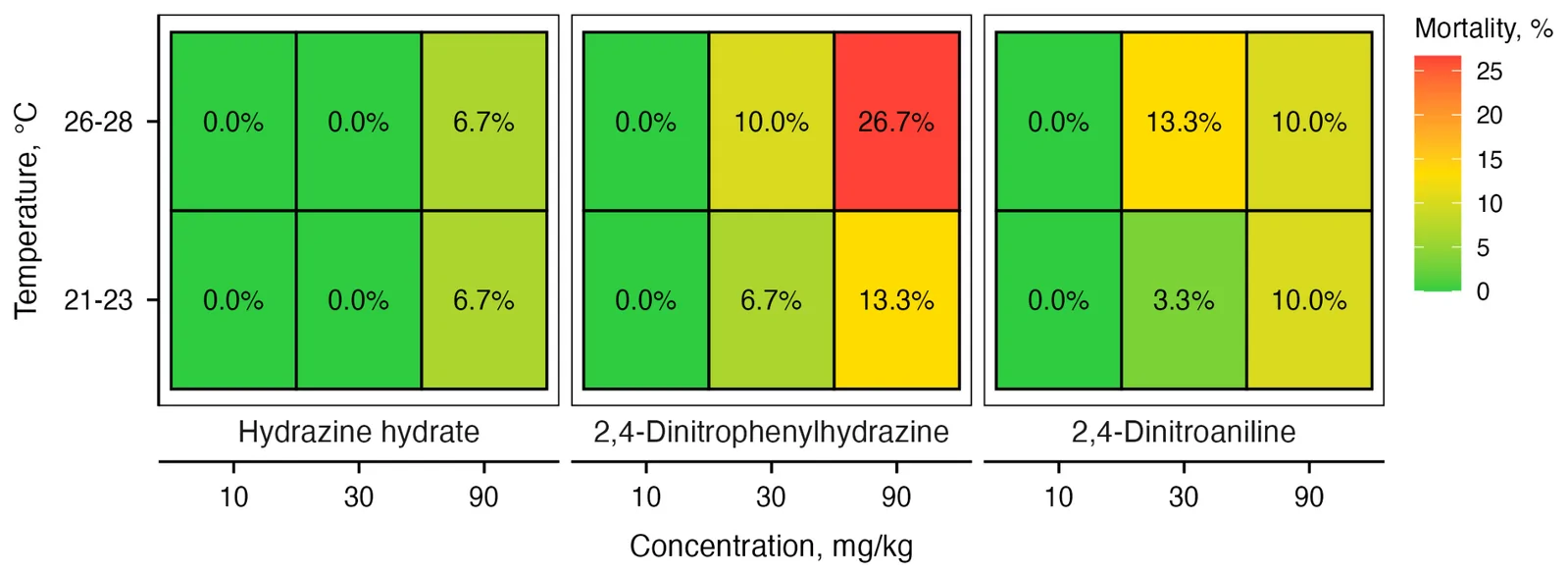
Mortality of *Tenebrio molitor* larvae after 5 days of exposure to the three studied compounds (hydrazine hydrate, 2,4-dinitrophenylhydrazine, 2,4-dinitroaniline) in the feed substrate at two temperatures. The heat map shows the percentage of mortality (numerical values in cells) with a color gradient from green (0.0% mortality) to red (26.7% mortality); n = 30 larvae per combination of compound, concentration and temperature.

**Figure 3:**
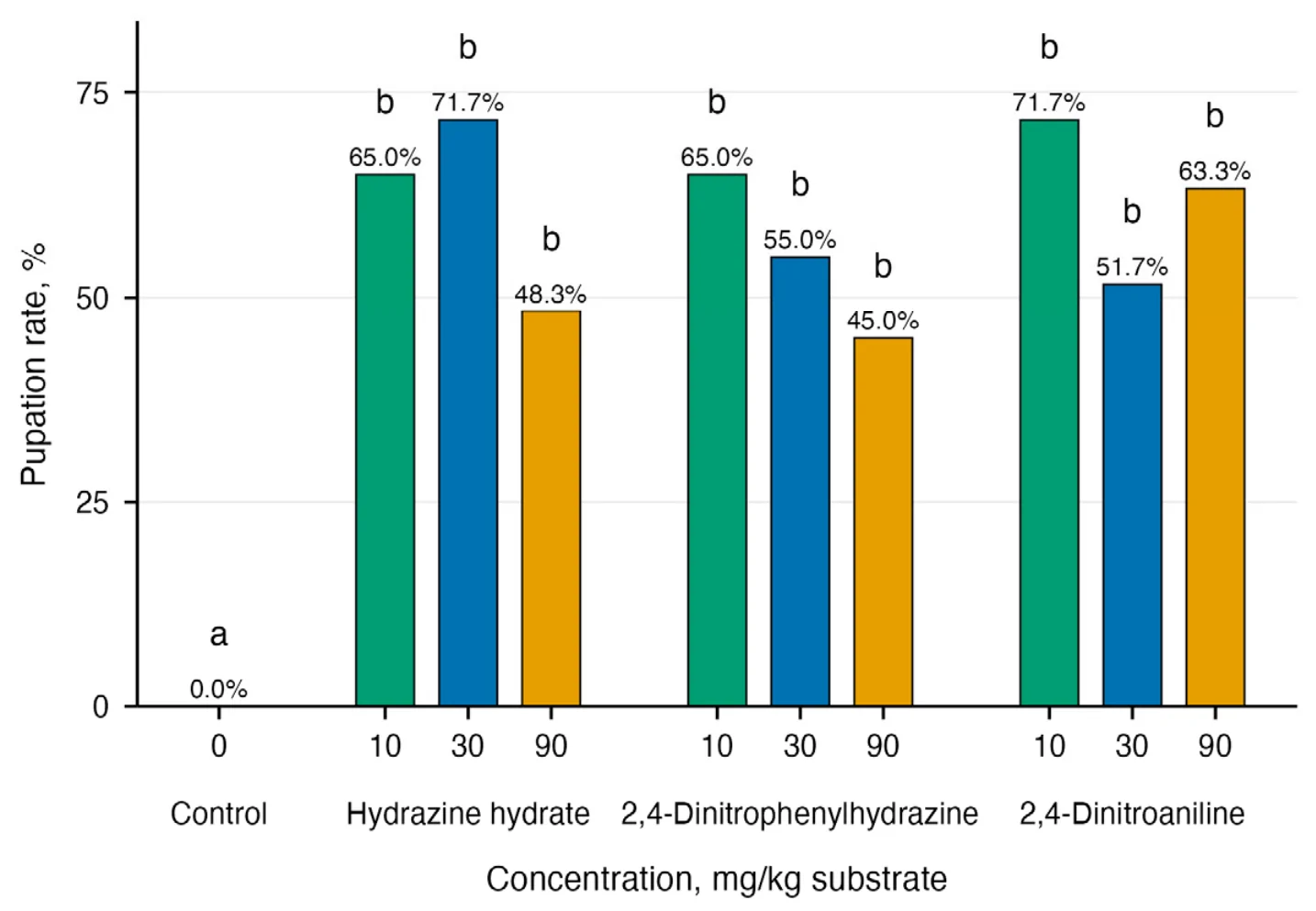
Pupation of *Tenebrio molitor* larvae during a 5-day exposure to hydrazine hydrate, 2,4-dinitrophenylhydrazine and 2,4-dinitroaniline in the feed substrate, pooled across both temperature regimes. Bars give the percentage of exposed larvae that pupated at each concentration, printed above the bar, with color repeating the concentration shown on the axis. Letters mark groups that do not differ statistically at α = 0.050, based on Fisher’s exact test for the control against the exposed larvae and a binomial mixed model among the exposed groups; n = 60 larvae per bar, in 6 containers of 10.

**Figure 4:**
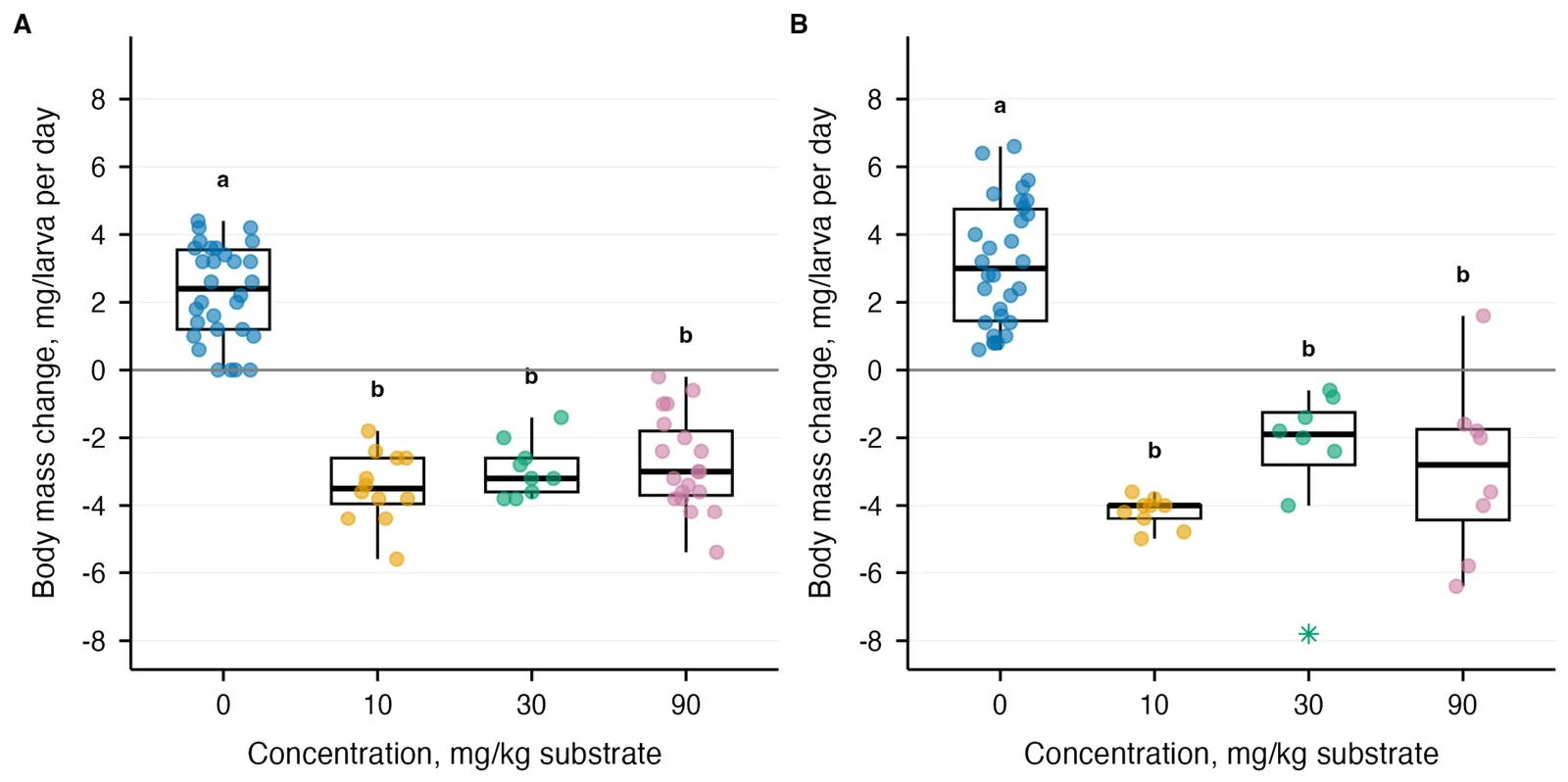
Effect of hydrazine hydrate on daily body mass changes in *Tenebrio molitor* larvae during a 5-day exposure: (**A**)—21–23 °C and (**B**)—26–28 °C; colored circles—individual larvae; asterisks (*)—outliers beyond 1.5× the interquartile range; the grey line marks zero mass change; letters mark groups that do not differ statistically at α = 0.050, from Tukey-adjusted contrasts of the linear mixed model; n = 125 larvae that did not pupate.

**Figure 5:**
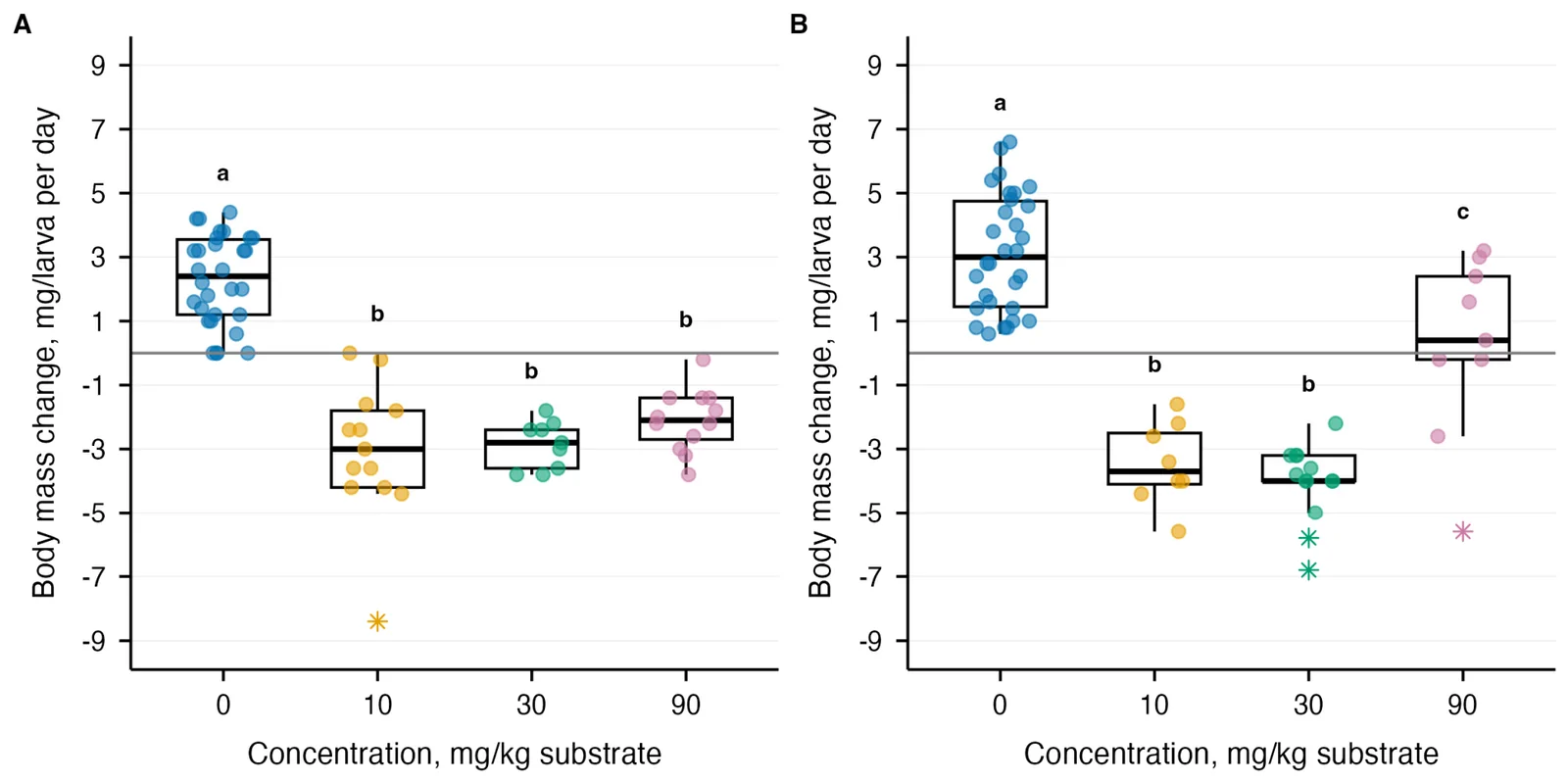
Effect of 2,4-dinitrophenylhydrazine (DNPH) on daily body mass changes in *Tenebrio molitor* larvae during a 5-day experiment: (**A**)—temperature 21–23 °C and (**B**)—temperature 26–28 °C (n = 124 larvae that did not pupate); see Figure 4 for an explanation.

**Figure 6:**
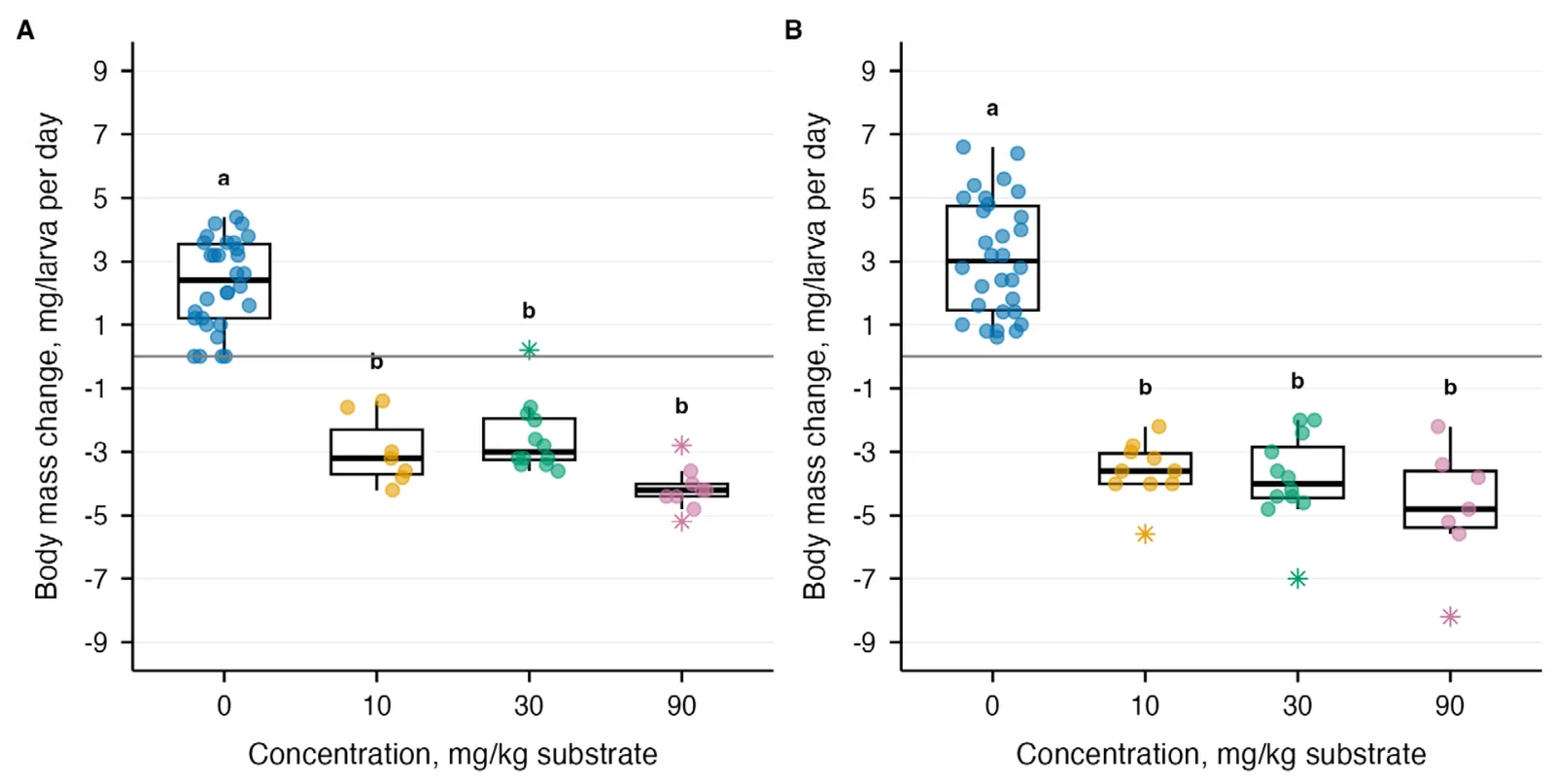
Effect of 2,4-dinitroaniline on daily body mass changes in *Tenebrio molitor* larvae during a 5-day experiment: (**A**)—temperature 21–23 °C and (**B**)—temperature 26–28 °C (n = 117 larvae that did not pupate); see Figure 4 for an explanation.

**Figure 7:**
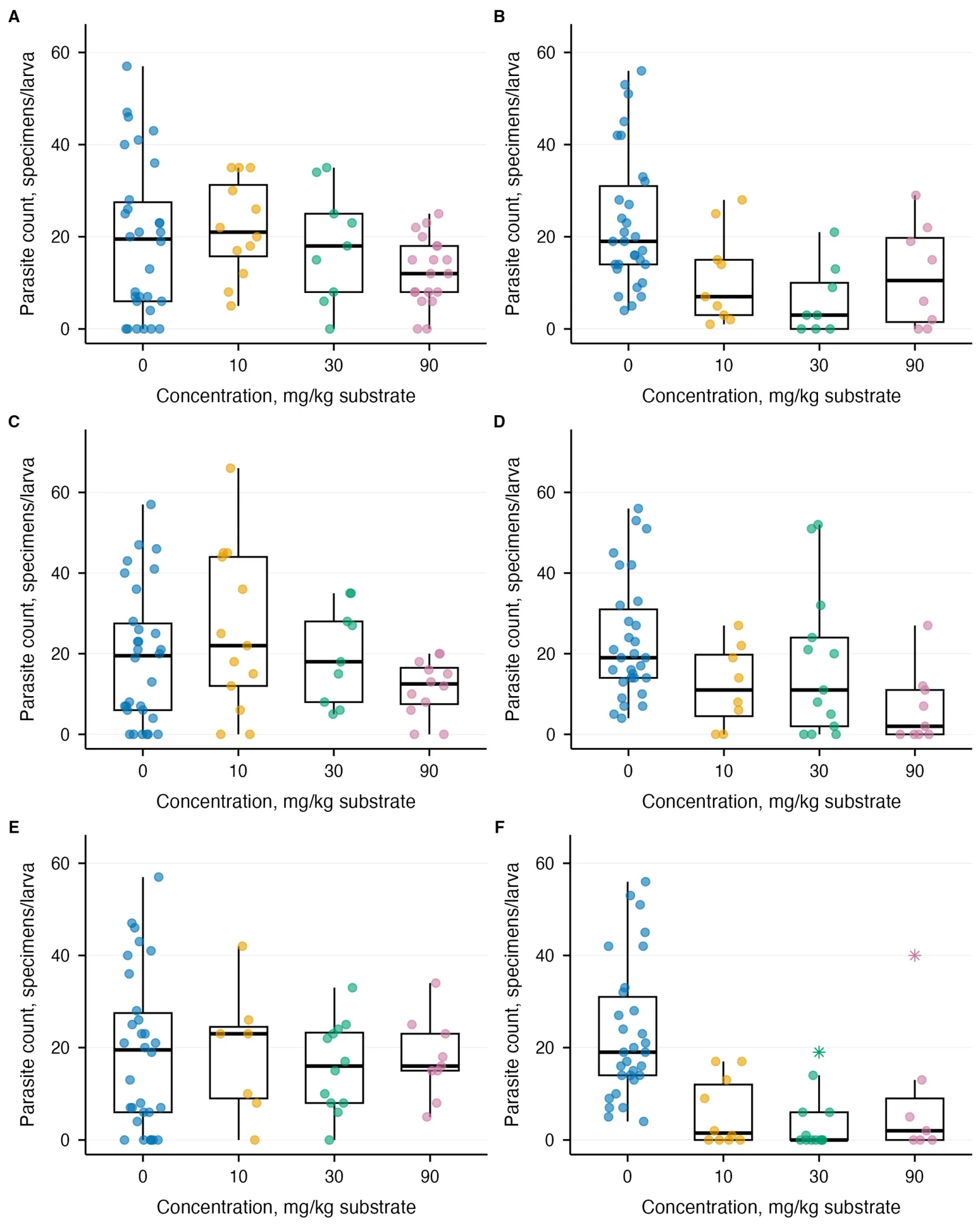
Total intensity of *Gregarina* spp. infestation, i.e., the sum of *Gregarina cuneata*, *G. polymorpha* and *G. steini* in specimens/larva, in *Tenebrio molitor* larvae during a 5-day substrate exposure: (**A**,**B**)—hydrazine hydrate (n = 125); (**C**,**D**)—2,4-dinitrophenylhydrazine (n = 124); (**E**,**F**)—2,4-dinitroaniline (n = 117), each including the same 60 control larvae; left panels—21–23 °C, right panels—26–28 °C; colored circles—individual larvae; asterisks (*)—outliers beyond 1.5× the interquartile range; all counts come from larvae that survived and did not pupate.

**Figure 8:**
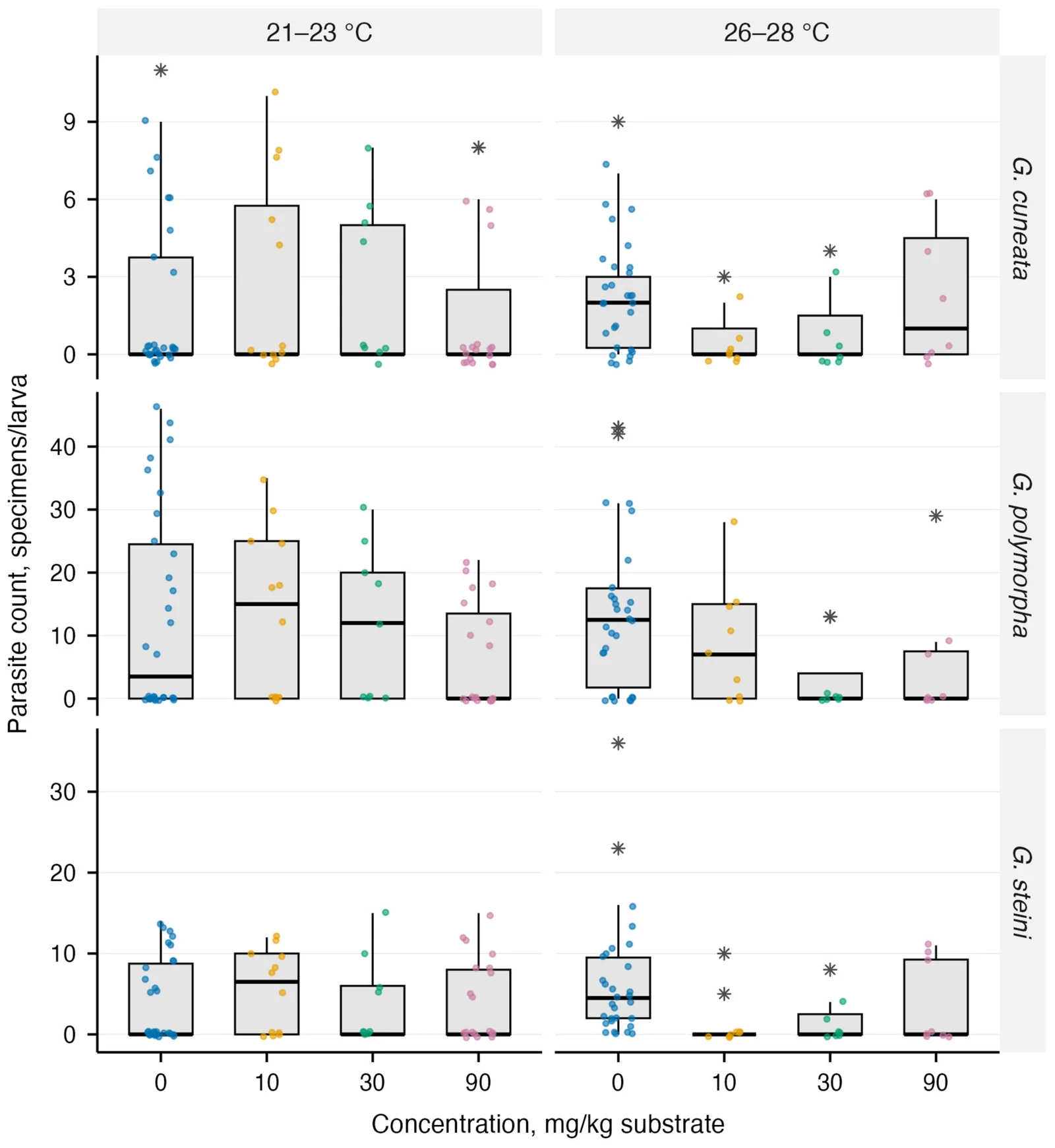
Effect of hydrazine hydrate on the counts of each parasite species (*Gregarina cuneata*, *G. polymorpha*, *G. steini*) in *Tenebrio molitor* larvae during a 5-day exposure: rows—parasite species, columns—temperature, left—21–23 °C, and right—26–28 °C; colored circles—individual larvae; asterisks (*)—outliers beyond 1.5× the interquartile range; the y-axis is scaled independently for each species; n = 125 larvae that survived and did not pupate.

**Figure 9:**
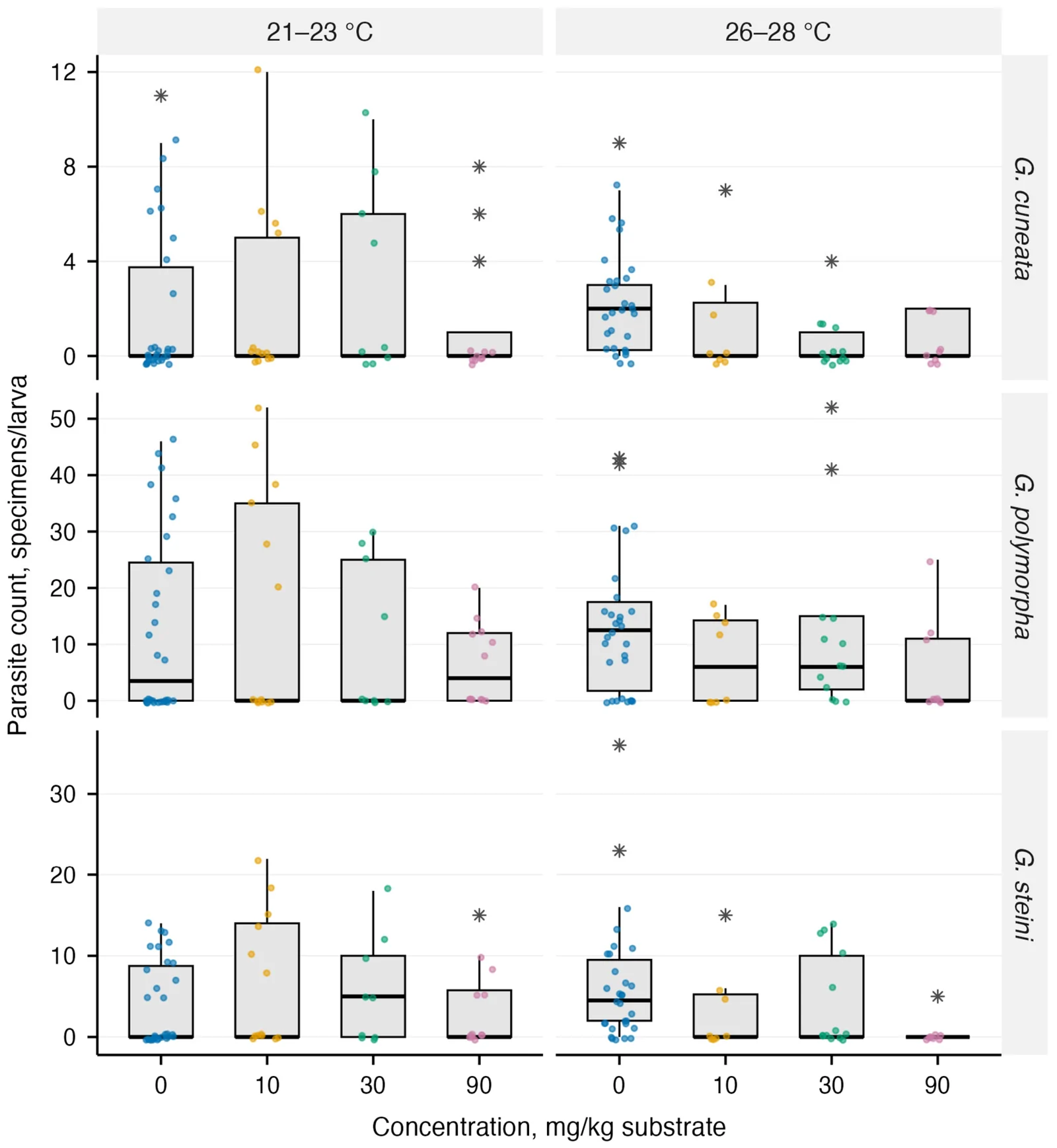
Effect of 2,4-dinitrophenylhydrazine (DNPH) on the counts of each parasite species in *Tenebrio molitor* larvae during a 5-day experiment: rows—parasite species, columns—temperature, left—21–23 °C, and right—26–28 °C (n = 124 larvae that survived and did not pupate); see Figure 8 for an explanation.

**Figure 10:**
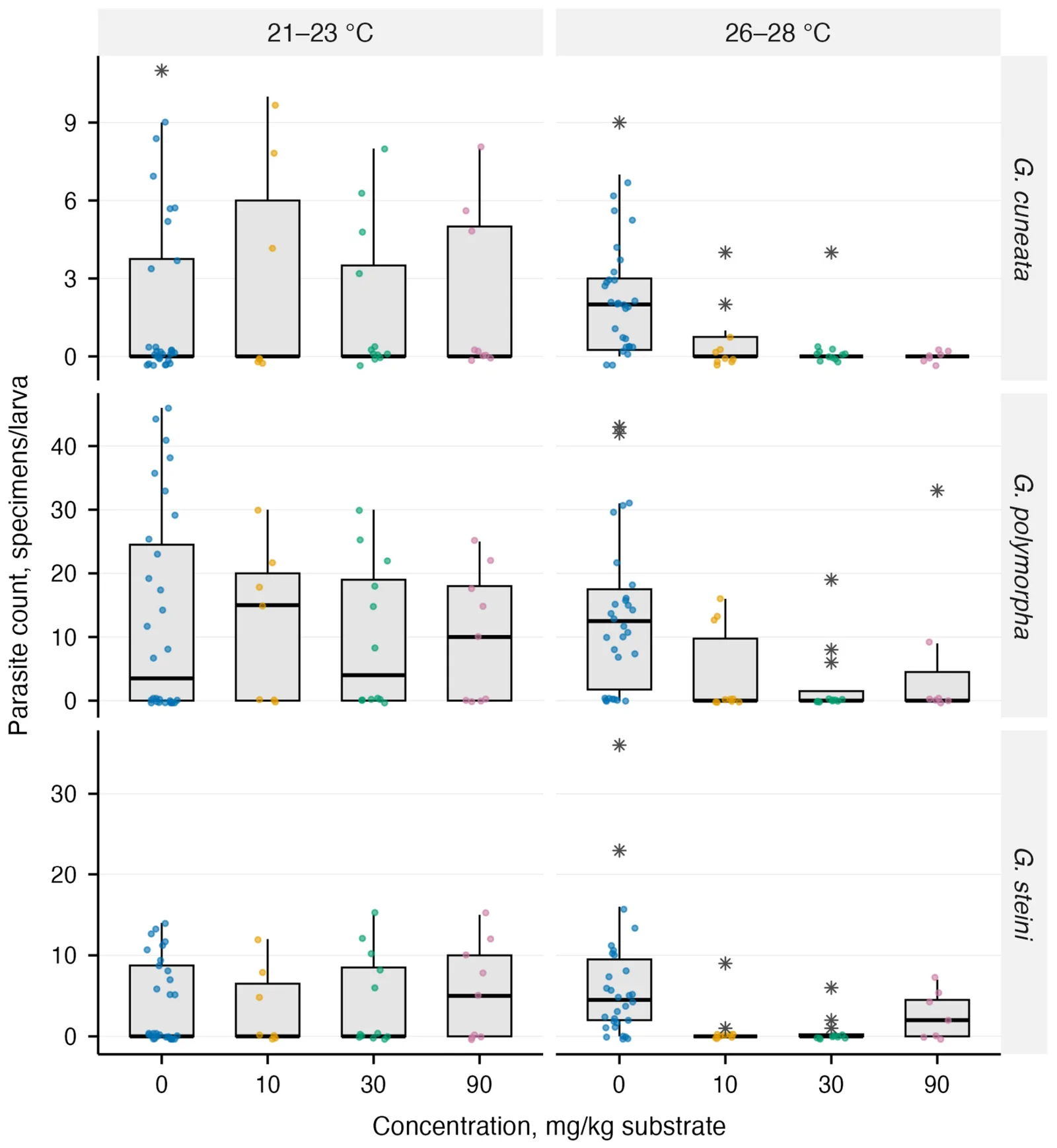
Effect of 2,4-dinitroaniline on the counts of each parasite species in *Tenebrio molitor* larvae during a 5-day experiment: rows—parasite species, columns—temperature, left—21–23 °C, and right—26–28 °C (n = 117 larvae that survived and did not pupate); see Figure 8 for an explanation.

**Table 1:** Odds ratios for the death of *Tenebrio molitor* larvae per additional 10 mg/kg substrate during a 5-day exposure to the three studied compounds, averaged over both temperature regimes. The 10 mg/kg step lies within the tested range of 10–90 mg/kg substrate, and a value above 1 denotes increasing odds of death; 95% CI—95% confidence interval; z—the Wald test statistic; *p*—the level of statistical significance; n = 600 larvae.

Compound	Odds Ratio	95% CI	z	*p*
2,4-Dinitrophenylhydrazine	1.41	1.25–1.59	5.46	<0.001
2,4-Dinitroaniline	1.30	1.13–1.49	3.79	<0.001
Hydrazine hydrate	1.19	1.02–1.40	2.16	0.031

**Table 2:** Proportion of *Tenebrio molitor* larvae that pupated during a 5-day substrate exposure to three hydrazine compounds. Data are pooled across the two temperature regimes (21–23 and 26–28 °C) and the three concentrations (10, 30 and 90 mg/kg substrate). The denominator is the number of larvae exposed rather than the number that survived because survival itself depends on the exposure; the proportion therefore expresses the probability of reaching pupation and incorporates larval mortality.

Group	n	Pupated	Proportion, %
Control	60	0	0.0
Hydrazine hydrate	180	111	61.7
2,4-Dinitrophenylhydrazine (DNPH)	180	99	55.0
2,4-Dinitroaniline	180	112	62.2
All treatment groups	540	322	59.6

**Table 3:** Differences in daily body mass changes between the nine treatment groups and the control in *Tenebrio molitor* larvae during a 5-day exposure to the three studied compounds, averaged over both temperature regimes; negative values denote mass loss relative to the control; 95% CI—95% confidence interval; d—Cohen’s d effect size; n = 246 surviving larvae that did not pupate.

Compound	Concentration, mg/kg	Difference, mg/Larva per Day	95% CI	d
Hydrazine hydrate	10	−7.13	−8.66 to −5.61	−4.98
Hydrazine hydrate	30	−5.77	−7.26 to −4.28	−4.03
Hydrazine hydrate	90	−5.96	−7.39 to −4.53	−4.17
2,4-Dinitrophenylhydrazine (DNPH)	10	−6.45	−7.93 to −4.97	−4.51
2,4-Dinitrophenylhydrazine (DNPH)	30	−6.52	−7.96 to −5.08	−4.55
2,4-Dinitrophenylhydrazine (DNPH)	90	−3.86	−5.30 to −2.41	−2.69
2,4-Dinitroaniline	10	−6.50	−8.04 to −4.95	−4.54
2,4-Dinitroaniline	30	−6.46	−7.92 to −5.01	−4.52
2,4-Dinitroaniline	90	−7.53	−9.06 to −6.01	−5.26

## Data Availability

The data presented in this study are available in GitHub at https://doi.org/10.15421/5126102 (accessed 12 September 2026).

## Supplementary Materials

The following supporting information can be downloaded at https://doi.org/10.15421/5126102 (accessed 12 September 2026).

## References

[1] Du J., Ren X., Zeng Y., Liu Y., Dong J., Yang S., Shi J., Liu B., Chen Y. (2026). Degradation dynamics and pathways of unsymmetrical dimethylhydrazine (UDMH) across contrasting soil matrices. Toxics.

[2] Dallas J.A., Raval S., Alvarez Gaitan J.P., Saydam S., Dempster A.G. (2020). The environmental impact of emissions from space launches: A comprehensive review. J. Clean. Prod..

[3] Velte J.S. (1984). Acute toxicity of hydrazine hydrate to the fathead minnow (*Pimephales promelas*) and daphnid (*Daphnia pulex*). Bull. Environ. Contam. Toxicol..

[4] Boyko O.O., Brygadyrenko V.V. (2026). Survival of nematode larvae under exposure to explosion-derived contaminants in war-affected environments. Biosyst. Divers..

[5] Rybalka D., Brygadyrenko V. (2026). Acute toxicity of TNT derivatives and hydrazine-based compounds from explosive and rocket fuel contamination to darkling beetles (*Tenebrio molitor* and *Opatrum sabulosum*). PeerJ.

[6] Broomandi P., Guney M., Kim J.R., Karaca F. (2020). Soil contamination in areas impacted by military activities: A critical review. Sustainability.

[7] Pichtel J. (2012). Distribution and fate of military explosives and propellants in soil: A review. Appl. Environ. Soil Sci..

[8] Palchykov V.A., Malaniia M.M., Tymoshenko K.I., Brygadyrenko V.V. (2025). Organic soil pollutants resulting from military activities. Biosyst. Divers..

[9] Khomiak I.V., Onyshchuk I.P., Kychkyruk O.Y., Vakerych M.M., Hasynets Y.S., Schwartau V.V. (2025). The impact of strike UAV explosions on soil acidity and vegetation dynamics. Biosyst. Divers..

[10] Radelyuk I., Zhakupbekova A., Zhumadildinova A., Kashtanov A., Baimatova N. (2025). Hidden contamination patterns: A stochastic approach to assessing unsymmetrical dimethylhydrazine transformation products in Kazakhstan’s rocket crash area. Toxics.

[11] Hu C., Zhang Y., Zhou Y., Liu Z.-F., Feng X.-S. (2022). Unsymmetrical dimethylhydrazine and related compounds in the environment: Recent updates on pretreatment, analysis, and removal techniques. J. Hazard. Mater..

[12] Leal Filho W., Eustachio J.H.P.P., Fedoruk M., Lisovska T. (2024). War in Ukraine: An overview of environmental impacts and consequences for human health. Front. Sustain. Resour. Manag..

[13] Solokha M., Demyanyuk O., Symochko L., Mazur S., Vynokurova N., Sementsova K., Mariychuk R. (2024). Soil degradation and contamination due to armed conflict in Ukraine. Land.

[14] Biyashev B., Drobitko A., Markova N., Bondar A., Pismenniy O. (2024). Chemical analysis of the state of Ukrainian soils in the combat zone. Int. J. Environ. Stud..

[15] Spencer P.S., Kisby G.E. (2021). Role of hydrazine-related chemicals in cancer and neurodegenerative disease. Chem. Res. Toxicol..

[16] Crosby M.E., Ciurlionis R., Brayman T.G., Kondratiuk A., Nicolette J.J. (2022). Exploring the molecular and functional cellular response to hydrazine via transcriptomics and DNA repair mutant cell lines. Environ. Mol. Mutagen..

[17] LeFort K.R., Rungratanawanich W., Song B.J. (2024). Contributing roles of mitochondrial dysfunction and hepatocyte apoptosis in liver diseases through oxidative stress, post-translational modifications, inflammation, and intestinal barrier dysfunction. Cell. Mol. Life Sci..

[18] Matsumoto M., Kano H., Suzuki M., Katagiri T., Umeda Y., Fukushima S. (2016). Carcinogenicity and chronic toxicity of hydrazine monohydrate in rats and mice by two-year drinking water treatment. Regul. Toxicol. Pharmacol..

[19] Jikamo S.C., Rao T.S., Shyamala P., Supriya S.S., Nayak S.R., Kadiyala N., Dokka W.T., Ravichandra M., Kishore M.V. (2025). Highly sensitive electrochemical detection of hazardous 2,4-dinitrophenylhydrazine using MgCo-TiO_2_/g-C_3_N_4_ heterostructure nanocomposites. RSC Adv..

[20] Giglio A., Vommaro M.L. (2022). Dinitroaniline herbicides: A comprehensive review of toxicity and side effects on animal non-target organisms. Environ. Sci. Pollut. Res..

[21] Kim S., Chen J., Cheng T., Gindulyte A., He J., He S., Li Q., Shoemaker B.A., Thiessen P.A., Yu B. (2023). PubChem 2023 update. Nucleic Acids Res..

[22] Sokolova I.M., Lannig G. (2008). Interactive effects of metal pollution and temperature on metabolism in aquatic ectotherms: Implications of global climate change. Clim. Res..

[23] Brai A., Poggialini F., Vagaggini C., Pasqualini C., Simoni S., Francardi V., Dreassi E. (2023). *Tenebrio molitor* as a simple and cheap preclinical pharmacokinetic and toxicity model. Int. J. Mol. Sci..

[24] Bordiean A., Krzyżaniak M., Stolarski M.J., Czachorowski S., Peni D. (2020). Will yellow mealworm become a source of safe proteins for Europe?. Agriculture.

[25] Lazurska V., Brygadyrenko V. (2024). Effects of organic xenobiotics on *Tenebrio molitor* larvae and their parasite *Gregarina polymorpha*. Biology.

[26] Rybalka D., Brygadyrenko V. (2025). Effects of toxic organic compounds on *Tenebrio molitor* and its parasite *Gregarina steini*. Biology.

[27] Gałęcki R., Sokół R. (2019). A parasitological evaluation of edible insects and their role in the transmission of parasitic diseases to humans and animals. PLoS ONE.

[28] Nocciolini C., Cucini C., Leo C., Francardi V., Dreassi E., Carapelli A. (2018). Assessing the efficiency of molecular markers for the species identification of gregarines isolated from the mealworm and super worm midgut. Microorganisms.

[29] Wolz M., Schrader A., Whitelaw E., Müller C. (2022). Gregarines modulate insect responses to sublethal insecticide residues. Oecologia.

[30] Schawang J.E., Janovy J. (2001). The response of *Gregarina niphandrodes* (Apicomplexa: Eugregarinida: Septatina) to host starvation in *Tenebrio molitor* (Coleoptera: Tenebrionidae) adults. J. Parasitol..

[31] Hong J., Han T., Kim Y.Y. (2020). Mealworm (*Tenebrio molitor* larvae) as an alternative protein source for monogastric animal: A review. Animals.

[32] Percie du Sert N., Hurst V., Ahluwalia A., Alam S., Avey M.T., Baker M., Browne W.J., Clark A., Cuthill I.C., Dirnagl U. (2020). The ARRIVE guidelines 2.0: Updated guidelines for reporting animal research. PLoS Biol..

[33] Cohen J. (1992). A power primer. Psychol. Bull..

[34] Clopton R.E., Janovy J., Percival T.J. (1992). Host stadium specificity in the gregarine assemblage parasitizing *Tenebrio molitor*. J. Parasitol..

[35] Harry O.G. (1967). The effect of a eugregarine *Gregarina polymorpha* (Hammerschmidt) on the mealworm larva of *Tenebrio molitor* (L.). J. Protozool..

[36] Valigurová A. (2012). Sophisticated adaptations of *Gregarina cuneata* (Apicomplexa) feeding stages for epicellular parasitism. PLoS ONE.

[37] Desportes I., Schrével J. (2013). Treatise on Zoology—Anatomy, Taxonomy, Biology. The Gregarines.

[38] Piepho H.P. (2004). An algorithm for a letter-based representation of all-pairwise comparisons. J. Comput. Graph. Stat..

[39] R Core Team (2024). R: A Language and Environment for Statistical Computing.

[40] Zeileis A., Kleiber C., Jackman S. (2008). Regression models for count data in R. J. Stat. Softw..

[41] Li M., Chen J., Wang Z.-Y., Wang F.-F., Dong L.-L., Pan J., Li M.-E., Yu L., Sang W. (2026). *TcALS* knockdown accelerates metamorphosis via insulin signaling in *Tribolium castaneum*. Insect Sci..

[42] Kodrík D., Bednářová A., Zemanová M., Krishnan N. (2015). Hormonal regulation of response to oxidative stress in insects—An update. Int. J. Mol. Sci..

[43] Sharma K., Mishra N., Shakarad M.N. (2020). Evolution of reduced minimum critical size as a response to selection for rapid pre-adult development in *Drosophila melanogaster*. R. Soc. Open Sci..

[44] Koul O. (2008). Phytochemicals and insect control: An antifeedant approach. Crit. Rev. Plant Sci..

[45] Shields V.D.C. (2023). Deterrents and their effects on the feeding behavior and sensory physiology of insects. Arthropods—New Advances and Perspectives.

[46] Rybalka D., Brygadyrenko V. (2026). The effect of ammonia on the host–parasite system *Tenebrio molitor* at different temperatures. Biology.

[47] Podlesnik J. (2025). Pupal development and adult acclimation temperatures influence the cold and heat tolerance in *Tenebrio molitor* (Coleoptera: Tenebrionidae). Insects.

[48] Herren P., Hesketh H., Dunn A.M., Meyling N.V. (2024). Heat stress has immediate and persistent effects on immunity and development of *Tenebrio molitor*. J. Insects Food Feed.

[49] Hryhorczuk D., Levy B.S., Prodanchuk M., Kravchuk O., Bubalo N., Hryhorczuk A., Erickson T.B. (2024). The environmental health impacts of Russia’s war on Ukraine. J. Occup. Med. Toxicol..

[50] Grygorieva O., Zaimenko N., Vergun O., Zhurba M., Goncharovska I., Golubkova I. (2025). Soil degradation caused by military activities in Ukraine: Challenges and prospects for ecological rehabilitation. Agrobiodivers. Improv. Nutr. Health Life Qual..

